# Non-COVID-19 UK clinical trials and the COVID-19 pandemic: impact, challenges and possible solutions

**DOI:** 10.1186/s13063-023-07414-w

**Published:** 2023-06-22

**Authors:** Ava Lorenc, Leila Rooshenas, Carmel Conefrey, Julia Wade, Nicola Farrar, Nicola Mills, Sangeetha Paramasivan, Alba Realpe, Marcus Jepson

**Affiliations:** QuinteT Group, Population Health Sciences, Bristol Medical School, University of Bristol, Canynge Hall, 39 Whatley Road, Bristol, BS8 2PS UK

**Keywords:** COVID-19, Pandemics, Randomised controlled trials, Patient selection, Recruitment

## Abstract

**Introduction:**

The COVID-19 pandemic impacted the operationalisation of non-COVID-19 clinical trials globally, particularly site and participant recruitment and trial success/stoppage. Trials which anticipate recruitment challenges may embed methods such as the QuinteT Recruitment Intervention (QRI) to help identify and understand the sources of challenges. Such interventions can help shed light on pandemic-related challenges. This paper reports our experience of the impact of the COVID-19 pandemic on conducting clinical trials with an embedded QRI, highlighting how the QRI aided in identifying challenges and potential solutions, particularly related to the site set-up and participant recruitment.

**Main body:**

We report on 13 UK clinical trials which included a QRI. Information is from QRI data and researchers’ experience and reflections. In most trials, recruitment was lower than even the lowest anticipated rates. The flexibility of the QRI facilitated rapid data collection to understand and document, and in some instances respond to, operational challenges. Challenges were mostly logistical, pandemic-related and beyond the control of the site or central trial teams. Specifically: disrupted and variable site opening timelines —often due to local research and development (R&D) delays— shortages of staff to recruit patients; fewer eligible patients or limited access to patients; and intervention-related factors. Almost all trials were affected by pandemic-related staffing issues including redeployment, prioritisation of COVID-19 care and research, and COVID-19-related staff illness and absence. Trials of elective procedures were particularly impacted by the pandemic, which caused changes to care/recruitment pathways, deprioritisation of services, reduced clinical and surgical capacity and longer waiting lists. Attempted solutions included extra engagement with staff and R&D departments, trial protocol changes (primarily moving online) and seeking additional resourcing.

**Conclusion:**

We have highlighted wide-ranging, extensive and consistent pandemic-related challenges faced by UK clinical trials, which the QRI helped to identify and, in some cases, address. Many challenges were insurmountable at individual trials or trials unit level. This overview highlights the need to streamline trial regulatory processes, address staffing crises, improve recognition of NHS research staff and for clearer, more nuanced central guidance on the prioritisation of studies and how to deal with the backlog. Pre-emptively embedding qualitative work and stakeholder consultation into trials with anticipated difficulties, moving some processes online, and flexible trial protocols may improve the resilience of trials in the current challenging context.

## Background

The COVID-19 pandemic has impacted clinical trials, particularly site opening, trial activation and participant recruitment. Although UK pandemic-related restrictions stopped in February 2022, the impact of the pandemic is ongoing and COVID-19 infections are still present. In the UK, the onset of the pandemic resulted in a rapid large-scale reorganisation of research staff, research infrastructures and research priorities [1]. Internationally, activation for trials (excluding trials of COVID-19 treatments/vaccines) was 57% of that expected pre-pandemic [2], and the pandemic increased trial stoppage (suspension, termination or withdrawal) [3]. A global survey of over 5000 trials using ‘Medidata’ found declining patient recruitment in March, April, and May 2020, with studies recruiting 65%, 79% and 74% of the figures for the same period the year before, respectively [4]. In November 2021, the number of UK commercial clinical trials had not returned to pre-pandemic levels, with 18% fewer commercial trials starting compared to 2019 [5]. Opening of and recruitment to UK commercial trials were lower than in other countries pre-pandemic and have declined even further since, only some of which is explained by the growth of clinical trials in China [5]. Although the impact of the pandemic varied depending on whether the service being investigated continued during the pandemic (e.g. emergency care) or not (e.g. elective surgery), even in priority areas such as cancer, fewer trials were initiated [6] and recruitment dropped dramatically [7, 8].

The QuinteT (Qualitative research integrated within Trials) team, at the University of Bristol, UK, comprises researchers who specialise in optimising recruitment and informed consent to randomised controlled trials (RCTs), particularly trials with anticipated or emerging difficulties with recruitment. We mainly use the QuinteT Recruitment Intervention (QRI), a complex mixed-methods intervention to understand RCT-specific recruitment challenges. QRIs collect detailed screening data, audio-recordings of recruitment appointments (conversations between recruiting clinician and patient), observational data of trial staff meetings and events, site surveys and qualitative interviews with trial recruiters and participants [9]. Analysis of this data includes exploring how clinicians convey equipoise and other challenges in communication between recruiters. We then work with the trial team to implement solutions to the challenges identified [10]. The QRI has successfully been used to identify common challenges to RCT recruitment and strategies to assess them [9, 11–18]. This paper reports our experience of the impact of the COVID-19 pandemic on conducting clinical trials with an embedded QRI, highlighting challenges and potential solutions, particularly related to the site set-up and participant recruitment. We also explore how an embedded qualitative intervention can make trials more resilient. Information was collated by the QuinteT researchers from notes from study meetings, site surveys, study reports and QRI data, supplemented by the researcher’s experience and reflections. Some trial managers and chief investigators provided additional clarification.

This paper capitalises on the broad experience of the QuinteT team to synthesise and provide an overview of issues across a range of trials to add to existing literature, which often reports on single trials, e.g. in the *Trials* special collection on the impact of COVID-19 (https://www.biomedcentral.com/collections/covidtrialsimpact).

## Main text

This paper reports trial progress up to mid-2022 for 13 multi-site RCTs with a QRI (see Table 1). Eight investigated surgical procedures, one chemotherapy, one medication, two dialysis and one prehabilitation prior to surgery. Six trials started recruitment pre-pandemic, six during the pandemic and one after pandemic-related restrictions were lifted (February 2022 in the UK). Trials ranged from small (5–100 patients/1–10 sites) to very large (over 5000 patients/100 sites). The impact of the pandemic on trial viability varied—four trials were critically affected by severe multiple issues, another five were struggling and four were minimally impacted. At the time of writing, three trials were closing early (trials 7, 8, 13), one has reached target (trial 6), others continued to recruit.

**Table 1:** The 13 trials

Trial	Condition	Treatment	Target sample size range^a^	Number of active UK sites (at time of writing)^a^	Timing of trial opening to recruitment^b^		
1	Renal	Dialysis	300–400	1–10		During pandemic	
2	Renal	Dialysis	500–600	20–30	Pre-pandemic		
3	Oncology	Surgery	None	10–20		During pandemic	
4	Neurosurgery	Surgery	200–300	1–10		During pandemic	
5	Orthopaedics	Surgery	400–500	10–20			Post-restrictions
6	Neurosurgery	Surgery	50–100	20–30		During pandemic	
7	Orthopaedics	Surgery	300–400	1–10		During pandemic	
8	Urology	Surgery	200–300	10–20	Pre-pandemic		
9	Oncology	Surgery	750–1000	50–100	Pre-pandemic		
10	Oncology	Chemotherapy	4000–5000	100 +	Pre-pandemic		
11	Hepatobiliary	Surgery	5000 +	50–100	Pre-pandemic		
12	Mental health	Medication	300–400	10–20		During pandemic	
13	Surgery	Others	2000–3000	1–10	Pre-pandemic		

^a^We have not given precise numbers as this would potentially identify trials

^b^Pandemic defined as from March 2020 to February 2022 when all legal UK restrictions were lifted

### Impact of the pandemic on recruitment

In 11/13 trials, participant recruitment was lower than expected for pandemic-related reasons, as low as ¼ of expected (Trial 2). There was no apparent association between studies set up before vs after March 2020 and their recruitment success. In two trials (1 and 5), recruitment was delayed, but participant numbers were recovering. Trials 5 and 12 were unusual in being able to pause during the pandemic but before recruitment began, and trial 12 planned to use online and remote recruitment before the pandemic broke out. In trial 10, whilst monthly recruitment was negatively impacted, some sites managed to stay open during the pandemic, keen to give patients the opportunity to participate as the trial intervention meant potentially avoiding hospital-based chemotherapy and thus reducing COVID risk.

Although data on reasons for poor recruitment are limited (partly due to limited staff capacity to provide data for the QRIs), trial staff cited disrupted site opening, fewer eligible patients, shortages of staff to recruit patients and intervention-related factors. Each of these topics is explored in detail below.

### Reasons for recruitment challenges

#### Disrupted site set-up

QRI data for all 13 trials suggested study staff felt the pandemic had disrupted site set-up, particularly trials that were set up/opened during, rather than prior to, the pandemic. It is however difficult to ascertain if site set-up took longer than expected—two trials cited rates of site set-up at around 50% of planned rates, and in two trials that paused some sites never reopened when they restarted. Detailed data were available from 5/13 trials (see Table 2). The mean time to site approval (from the trial starting to the site open to recruitment) ranged from 4.8 to 20.7 months. However, similarly long set-up times for sites have been reported since 2008, with 52% of sites taking at least 6 months to be activated [19] and figures from 2014 and 2020 of average set-up times of 9.7 months [20] and 6 months [21].

**Table 2:** Site approval time

Trial	Mean time to site approval	Range of time to site approval
Trial 1	14 months	Data not available
Trial 4	4.8 months	0 to 8.4 months
Trial 5	20.7 months	16.8 to 24.9 months
Trial 6	7.9 months	3.9 to 12.9 months
Trial 7	5.5 months	3.2 to 12.6 months

Although we have limited data on reasons for site set-up disruption, it was often perceived as due to a delay in receiving site research and development (R&D) approval (five trials), but also clinical and research staff shortages (four trials; explored below), prioritisation of COVID-19 studies (three trials) and lack of research capacity (barrier to opening in 4/6 sites in trial 13). In two trials (trials 1 and 13), site opening was impacted by space/facilities/resources needed to provide the study intervention being used for COVID-19 services. Slow R&D approval was attributed to difficulties recruiting staff to vacant R&D posts and the huge additional burden of work from COVID-19 trials, particularly when paused trials restarted [22]. Data suggested that R&D departments were overwhelmed, with a 60% increase in studies in set-up in December 2021 compared to March 2019 [23].

Disrupted site set-up was perceived as majorly impacting recruitment, especially for the two trials of rare conditions where each site was only recruiting a few participants.

There was also wide variation between sites in different geographical areas *within* trials (which were all multi-site) in time to site approval (see Table 2), as previously identified by NHS England who found variation of up to 9 months [24]. Although data on reasons are limited, it is likely due to varying capacity and capability, staff, administrative and resource availability, prioritisation of trials [19, 25, 26] and possibly R&D departments waiting for healthcare services to return to normal. These geographical disparities may exacerbate existing health inequalities, for example, slower access to services such as elective care in more deprived areas [27]. In addition, varying research activity levels between sites may have repercussions for patient outcomes, which are thought to be better in research-active hospitals [25, 26] and for patient access to opportunities to take part in research.

#### Staffing issues

Nine trials reported pandemic-related staffing issues due to redeployment, prioritisation of COVID-19 care or research and COVID-19-related staff sickness/isolation/stress. This is confirmed by other papers, which also mention social distancing and working from home [1, 28, 29]. Hillman suggests the pandemic put further pressure on an already fragile system, where research is not fully embedded in the NHS nor integrated into staff roles, with little protected research time [5].

The main issue was reduced capacity of research staff (including research nurses (RNs)), but also clinical staff and clinical trials units (CTUs). RNs were busier (for example, in trial 2, the number of trials each RN was working on had increased in almost half the sites) and had less availability (for example around half of the RNs were unavailable due to redeployment on COVID-19 trials in the early stages of recruitment to trial 3). There was also a high degree of uncertainty, especially in the early pandemic stages, making trial process planning difficult. Staff shortages impacted site opening, participant recruitment (for example, not pursuing patient consents) and engagement in the QRI. The extent of staffing issues varied across sites, possibly related to site involvement in COVID-19/vaccine research, amongst other factors. One trial (trial 1) reported CTU staff shortages due to a halt in new staff recruitment, furlough and staff leaving. Two trials (trials 6 and 12) highlighted challenges due to reduced site staff interaction, e.g. multi-disciplinary team meetings moving online and different locations for research and clinical staff.

Recently, reports are emerging in the UK and the USA of a ‘crisis’ in the recruitment and retention of clinical trial staff [30, 31]. This seems to be due to the high workload and trial complexity, particularly for clinical research specialists (e.g. CTU staff, RNs and investigators), doctors and nurses [32]. In the UK, the NIHR portfolio is larger than ever, due to ongoing COVID-19 research and delayed trials restarting or being set up [22]. Staff report feeling stressed and exhausted [1], morale is low and there is a lack of support and opportunities for promotion [31].

#### Fewer eligible patients and lack of patient contact

Many of our trials recruited from hospitals, so reduced numbers of patients attending due to COVID-19 risk impacted trial recruitment, as previously reported [33, 34]. For two trials, recruitment was impacted by a reduction in patient contact due to appointments moving to off-site or remote delivery. This may be a long-term change, with reduced patient contact still reported by between half and two-thirds of sites in November 2021 (trials 2 and 13). In three trials patients were also moved, or chose to move, to private providers. This meant the trial staff struggled to access patients or data (reported by 2/7 sites in trial 13). Another issue was a shifting in personal priorities for patients, especially those with less serious conditions. Other papers have also reported poor recruitment due to the lack of patient contact and being unable to take paper consent [35, 36].

Two trials also reported fewer patients fitting eligibility criteria, e.g. the condition progressed during lockdown therefore patients became ineligible (trial 5), or fewer patients had the eligible injury during lockdown (trial 4). In trial 3, there was a practice change accentuated by pandemic restrictions, leading to fewer eligible patients.

#### Intervention delivery and outcome assessment

Trials of/related to elective procedures were particularly impacted by the pandemic—long waiting lists were common, due to procedures being deprioritised and low clinical capacity (staff and facilities/equipment)—an issue in 6/9 sites in trial 2. This reduced the number of available patients but also often resulted in changes to pathways which reduced opportunities for recruitment appointments, e.g. unpredictable waiting lists in trial 13, with surgeries arranged at short notice limiting the time for the pre-surgery intervention. It was challenging for clinicians to prioritise research against these competing pressures [5].

For some procedures (three trials), pandemic restrictions limited their delivery [35] or impacted recruitment, e.g. home-based interventions (trial 2) or aerosol-generating baseline assessment procedures necessitating extra ventilation time between patients so there were fewer patients per session (trial 13). In another trial, the intervention was reportedly more commonly offered to patients by some sites during the pandemic, reducing the pool of eligible patients for recruitment (trial 10) [35].

In four trials, the pandemic affected outcome measurement due to the lack of patient contact (discussed above), treatment delays impacting outcome measurement timing, or lack of capacity for developing online data collection technologies due to high demand.

### Success of embedded QRI

Many of the pandemic-related challenges identified were logistical barriers which also limited the QRIs' ability to explore hidden challenges to recruitment [37]. However, having the QRI integrated at trial design facilitated rapid data collection to understand and document the challenges. Using interviews with the study staff and analysis of screening data, the QRI was able to provide detailed data on barriers and challenges, which may inform decisions about prioritisation in trial portfolios. QRIs rapidly adapted, and many added opportunities for engagement with staff (RNs, principal investigators (PIs), recruiters) to identify emerging challenges. This engagement included study/site staff meetings (to update trial teams on adaptations to trial procedures/recruitment/clinical updates, energise local research teams and troubleshoot site issues), staff training on identifying and discussing the study with potential participants, webinars, newsletters, prize incentives and meetings with RNs. In five trials (trials 2, 6, 7, 11, 13), the QRI adapted to start staff training before sites opened. Some QRIs introduced new methods of data collection, e.g. collecting data on issues from recruiters by email or survey.

### Solutions implemented

Although many challenges were beyond the control of trial teams/units, some trials attempted to mitigate challenges, often in response to QRI findings. This was through extra engagement opportunities for staff and R&D departments, amended study protocols and requests for additional resourcing.

The staff engagement and training integral to the QRI demonstrates the benefits of consultation with and involvement of stakeholders (in assessing both the potential impact of the pandemic and possible solutions) in successfully maintaining trials during the pandemic [38]. Wider engagement beyond the study team and patients may also be beneficial, including with funders, collaborating research centres and health systems [38].

Protocol changes included primary outcome timing reflecting delays to surgery (trial 11), switching to online patient contact, e.g. online/postal consent and/or data collection, removing extra visits and medication by post. Many changes are likely to be sustained, in particular, moving towards digitisation [39, 40]. Other positive changes were staff adaptability, improved teamwork and innovations to interventions [38]. Some studies suggest electronic/online recruitment and consent may improve recruitment rates [41], trial diversity [42] and efficiency [43], even during pandemic lockdowns [44], although possibly result in lower conversion rates than offline strategies [41]. Online/remote study meetings, e.g. site initiation visits, are beneficial (better attendance, lower costs, can be more frequent) and unlikely to impair setup times, screening, recruitment or data collection [45]. Online recruitment may widen existing digital inequalities [46] but conversely may help to recruit underserved groups [47]. Online data collection is potentially more acceptable to participants, even for clinical measures [43].

Many of the pandemic-related challenges were exacerbated by inflexible trial protocols unable to adapt to using digital methods. Protocols using pragmatic trial designs and a variety of recruitment options may have helped [48]. The POWER study is a good example of a redesigned and successfully recruiting trial where the comparator was switched prior to site opening from surgery to waiting list to accommodate anticipated recruitment challenges resulting from pandemic-related delays to surgery [49].

Some sites sought additional resourcing, e.g. requesting recruitment extensions from funders, recruiting new staff or promoting the NIHR Associate Principal Investigators’ scheme (a 6-month in-work training opportunity for healthcare practitioners starting their research journey) to enable study workload pressures on site clinicians/research staff to be alleviated [50].

### Recommendations

This overview of 13 clinical trials has demonstrated the range of ways the pandemic has impacted on recruitment of trial participants and sites.

We have shown that an embedded qualitative intervention (in this case the QRI), opportunities to engage with trial teams, moving online and flexible trial protocols are beneficial in mitigating pandemic-related challenges. This highlights the value of trials likely to face difficulties embedding qualitative work and stakeholder consultation around challenges faced and possible solutions, for both pandemic and non-pandemic-related reasons. Although some of the issues identified, particularly the lack of patients, are likely to improve with the lifting of pandemic-related restrictions, the site/trial set-up disruption, capacity and staffing issues are ongoing and urgently need to be addressed.

The multi-site studies reviewed highlight the wide variation in study set-up times and ongoing disruption to set-up, mainly attributed to R&D. Studies exploring whether this is pandemic-related are warranted. Kolstoe and Carpenter highlight that although the new HRA (Health Research Authority) process recommends local R&D offices only address local capacity and capability issues, hospital sites often create new local processes, causing delays [51]. The system is improving [52]; however, further suggestions include deadlines on the R&D process, researcher access to data on local R&D performance, and fewer forms to complete [24, 53, 54]. There are opportunities to improve the system’s efficiency especially in these times of innovation and increased connectivity [55]. These changes may help to speed up and standardise the site set-up process [24, 54] and bring down timescales for study set-up in the UK in line with other countries [5]. Although unusual, the RECOVERY trial has shown how it is possible to rapidly set up a UK clinical trial if processes are streamlined and centralised [56]. The Department of Health and Social Care (DHSC) stated in September 2022 that they are ‘working closely with the NHS R&D community to identify ways to support them in tackling the current situation’ [23]. The UK government’s 2021 plan for delivering NHS clinical research now covers five areas including making the process streamlined, efficient and innovative, enabling through using data and digital tools, and a sustainable and supported research delivery workforce [57]. However, there is clearly a need to increase R&D capacity.

One suggestion to address the capacity issues of RNs and ‘crisis’ in the clinical trial staff recruitment/retention, is more opportunities for recognition of RNs’ skills and experiences and validation/progression support via a nationally accredited scheme similar to the NIHR associate PI scheme. We also support Mitchell et al.’s recommendations for greater recognition of the role of the clinical trial manager, improving the role via flexible working and longer contracts, and making efforts to attract people to the role [30]. The DHSC is working on a ‘cross-sector research workforce plan’, including additional investment in the research workforce from 2024 [23], which may help in the longer term.

That the pandemic differentially affected certain clinical areas and types of interventions may lead to a disparity in available treatments between clinical areas, for example, in neuro-oncology it is anticipated that the availability of new, potentially life-extending treatments has been reduced due to the premature closure of clinical trials in this area [58].

The use of routine health care for research and analysis, and embedding research in NHS practice, can help overcome barriers and is an important focus for both the government and NHS [27], included in the UK government’s 2021 plan for clinical research in the NHS following the pandemic [57]. Resourcing is needed to help clinical staff and hospital management embed trials in their settings [20]. Hillman provides the example of COVID-19 studies where every single COVID-19 patient was considered for enrolment, with great success [5]. Research needs to be integrated into staff roles, with protected research time [5].

In early 2022, the NHS acknowledged the challenges facing the NHS research portfolio, including staffing, and made plans to ‘take firm action on studies that are struggling to deliver’ [59], with the Reset programme in September 2022 confirming the closure of studies not feasible within current resources [23]. Guidance on the prioritisation of studies to consider for closure is however vague and largely based on crude recruitment figures rather than factors such as the priority of the research question or health condition or analysis of the specific barriers and how effectively these could be overcome.

Many challenges were insurmountable at individual study, or even trials unit level, yet there has been a paucity of clear guidance from central bodies on how to overcome them. As long as new trials continue to be considered for funding, the backlog of research will not be cleared. Strategic decision-making is needed on priorities for UK NHS clinical research and how this translates to a local R&D level. The NIHR states that it is ‘vital’ that new studies continue to open [23], but this appears unrealistic unless moves are made to streamline the regulatory process, increase R&D capacity and trial staffing, and give greater recognition to NHS staff’s contribution to research.

Embedding qualitative interventions into trials provides the opportunity to quickly identify and help address recruitment challenges. Such interventions may improve a trial’s resilience when facing the challenges to recruitment identified in this paper.

## Conclusions

This analysis of 13 trials has highlighted the range of ways the pandemic continues to impact the success of UK clinical trials. Pandemic-related challenges included staffing issues, local R&D-related disruption to sites opening, prioritisation of healthcare and difficulty accessing patients. Many of these were insurmountable at individual study, or even trials unit level, highlighting the need for central action on the staffing/capacity crises in research and R&D and the ever-increasing NHS trial portfolio, and clear guidance on prioritisation of studies.

The QRI was able to adapt to understand, document and, in some instances respond to, challenges and provide data potentially useful in prioritisation exercises within trial portfolios. Trials, especially those likely to face difficulties, should consider the value of embedding methods to address recruitment difficulties, using stakeholder consultation, routine data, online tools and flexible pragmatic protocols.

## Data Availability

Not applicable.
